# Foreign Bodies in the Ear

**Published:** 1868-06

**Authors:** Henry L. Shaw


### SELECTED ARTICLES.

### ARTICLE VIII.

### *Foreign Bodies in the Ear.*

By Henry L. Shaw, M. D.

A foreign body in the ear is always a source of anxiety to the friends of patients; and although its removal, if accomplished in season, is quite easy, it is often by delay rendered very difficult. Most of the foreign bodies met with in the ears of children are put in while at play, and are often forgotten. With adults their introduction is almost invariably due to the use of extemporaneous ear picks for the relief of the intolerable itching in chronic inflammation of the dermoid layer of the external auditory canal.

The ear is more tolerant of foreign bodies than is generally supposed. Cotton, which, from a belief in its virtues, is frequently introduced into the meatus, would often remain for an indefinite time, if the patient was not admonished by the increasing deafness to seek relief. Toynbee speaks of a dissection where cotton, which had probably been in the ear for years, produced absorption of the bony meatus. We can recall several cases where it remained for many years, unknown to the patients. Other foreign substances may be carried the same length of time. In a late number of the *Lancet* is the report of a case, where a piece of slate pencil was left in the year for over forty years. In one of our own cases, a stone, the size of a pea, remained in the canal for years before trouble was produced, and it was then caused by attempts at removal. Still another case was that of a playmate, who carried a bean in his ear for twenty years, with no bad effect, except slight deafness.

In our own experience the following substances have been met with; beans, cotton, slate pencils, peas, maggots, cockroaches, beads, glass, crockery, shells, paper, pins, ivory, teeth of combs, stones and seeds.

The amount of trouble produced by foreign bodies in the ear depends upon their nature, position and size. Hard, smooth substances, and those not easily affected by moisture, produce far less trouble than those of softer material, which are readily expanded.

At about the middle of the external auditory meatus the canal is angular. This change in its course serves somewhat as a check to the passage of foreign bodies. It is in this part of the canal that they are apt to lodge, and may remain for years before producing any injury. In works on anatomy the external meatus is described as being narrowest at the middle. The meatus, just before it reaches the membrana tympani, is somewhat expanded, as is also the entrance. With the exception of this dilatation at the ends, its diameter is quite uniform. A casual glance might lead one to suppose that there was considerable narrowing at the angle, but on straightening the meatus this apparent narrowing will disappear. An examination of the casts at the Warren Museum, taken by Dr. R. M. Hodge, confirms the above statement.

The symptoms caused by the presence of a foreign body, depend very much upon its position. When imbedded in wax, as is often the case, or fixed in the walls of the meatus, it will not be likely to cause serious trouble. Not, so, however, if it is at the bottom of the canal, in contact with the membrana tympani, or pressing upon it. Such a case is usually attended with giddiness, and a feeling of fulness of the head; which, if the foreign substances, are allowed to remain, may be followed by convulsions and even a fatal result. One would suppose, from the fact that casts of hardened cerumen are occasionally taken from the lower half of the canal, that the membrana tympani would readily tolerate the presence of a foreign body. When pressure is applied over that portion against which the handle of the malleus rests, it is attended with pain and marked cerebral disturbance. The same is true of the rest of the drum, but in a less degree. Besides the injurious effects above alluded to, the pressure of a foreign body on the membrana tympani is very likely to be followed by ulceration and perforation of that membrane, and organic changes in the tympanic cavity, which will seriously affect its integrity. Many cases of internal otitis owe their origin to this cause. We can recall two cases of the kind; in one of which the suppurative process was arrested by the removal of a piece of slate pencil, which protruded into the tympanum; in the other, the suppuration was undoubtedly prolonged from the presence of a glass bead in the tympanum.

When a foreign body is so large as to fill the whole diameter of the auditory canal, and press with considerable force upon its walls, it will almost invariably excite acute inflammation. In some of these cases the swelling is so great as to completely close the entrance of the meatus; rendering even an exploration impossible. When in this inflamed condition, the ear will be found to be very sensitive. The use of the speculum auris at this time will give rise to excruciating pain, and will be likely to be followed by considerable hæmorrhage. Under these circumstances all attempts at removal should be deferred, until the acute symptoms have subsided. Great relief will often be afforded by the application of leeches in front and below the external meatus, warm fomentations, etc. Occasionally, when suppuration begins, there will be a spontaneous discharge of the foreign substance.

In most cases foreign bodies are lodged in the angular portion of the canal; the exceptional cases being those where, from unsuccessful attempts at removal, they have been pushed through the membrana tympani, or where that membrane, from previous inflammation, or ulceration induced at the time by the pressure of the foreign bodies, has been perforated and has allowed them to pass beyond it. One would suppose that it would be impossible for a judicious practitioner to produce this result. This accident is, however, not uncommon, and can doubtless in most cases be traced to attempts at removal with instruments when the ear was poorly illuminated.

It is rare for foreign bodies to remain long in the tympanic cavity without producing serious symptoms. These will be modified somewhat by the nature of the substance, and the condition of the tympanum. If this has been previously disorganized by inflammation, as in most cases of otitis interna less trouble will probably ensue, than when it is in its normal condition. Beans and peas, the foreign bodies most frequently met with in the ear, are, from the facility with which they swell, most likely to produce fatal results. Undoubtedly in some cases the fatal result is due to the violent manipulations to which the ears have been subjected by the friends of patients, or to their not having consulted the surgeon, until inflammation and swelling have ensued, which rendered their removal extremely difficult or perhaps impossible.

When a patient is presented with a suspected foreign body in the ear, it is of great importance to examine thoroughly the auditory canal: much useless syringing may thus be avoided. By the improved method of Troeltsch this examination is possible at all times, and brings to view the whole of the meatus, and if necessary the tympanum.

Too much cannot be said in favor of the syringe for the removal of foreign bodies, of whatever kind, from the ear. As a rule it will be found successful; the exceptional cases are indeed very rare. Most authors agree as to its great advantages over all other instruments. Yet, to judge from the cases presented at the Infirmary, one is lead to believe that practically it is not much relied upon by the profession. With the syringe, accidents which sometimes attend the use of other instruments are avoided, as it is almost impossible with it to injure the surrounding parts. When the ear is well illuminated a foreign body may often be removed with instruments much more quickly than with the syringe, yet there is more risk; and the attempt, if unsuccessful, may, by injuring the walls of the canal, render removal of the substance by the syringe more difficult.

In this connection it may be well to speak of the manner of syringing an ear. Although generally considered an easy matter, it is often, from the non-observance of certain precautions, very ineffectual. The most important precaution is to straighten the canal, which, as is well known, is readily effected by pulling the external ear upward and backward with the left hand, while the right is free to use the syringe. By so doing we avoid putting the nozzle of the syringe into the external meatus, and thus frequently save the patient much pain, at the same time are enabled to act directly upon the foreign substance. The choice of a syringe is a matter of less importance; any one having a tightly adapted piston will usually succeed very well. The small two ounce rubber syringes, the pistons of which are generally accurately fitted, will be found the most reliable and convenient. The water used (which should be quite warm and pure) ought to be injected with very slight force at first, afterward the force may need to be considerably increased. The bursting of bubbles of air in the external meatus gives rise to very unpleasant sensations. This can generally be avoided by using a good syringe, and taking the precaution to fill it very slowly, so that no air shall be sucked up.

The facility with which a foreign body can be syringed from the ear depends somewhat upon its position, and very much upon the material. If it has passed but a short distance into the passage, a few syringesful will often be sufficient. Not so, however, if it is at the bottom of the canal, or impacted. Then the syringe may require to be used many minutes. Hard, smooth substances, as stones, beans, ect., are dislodged more readily than those of softer material, as paper, cotton, etc.

Foreign bodies sometimes become quite firmly attached to the walls of the canal, as in the interesting case reported by Dr. E. H. Clarke, where a bullet fixed in the bony meatus was removed by pressing upon it a strip of adhesive plaster, and then heating it by means of a convex lens until it adhered to the bullet. Should the symptoms admit of delay in these cases, the removal of foreign bodies may well be deferred, the passage being frequently filled with tepid water, until they are sufficiently loosened to allow their easy removal with the syringe.

Sometimes the foreign substance so completely plugs the meatus as not to allow the water to pass behind it. This, however, can only be ascertained by trial with the syringe. Many cases when examined by the speculum appear to be in this condition, but on using the syringe the foreign bodies are readily discharged. If, after continued syringing, the foreign substance is not removed, its position can sometimes be changed by the pointed end of a curette, or probe, when the syringe can again be used with greater probability of success. Only a very slight change in the position of a body is usually sufficient to ensure its removal with the syringe. Sometimes, however, the syringing has to be continued for a long time before it is successful.

With infants and young children great difficulty is often experienced in preventing violent movements of the head during the attempt at removal. An effort to straighten the canal even may be followed by a change in the position of the patient's head. When the passage is inflamed, the pain attending the removal may be very severe. Under these circumstances the use of ether will be found not only of great advantage, but frequently indispensable.

Cases requiring the exclusive use of instruments, are very rare. A most thorough trial of the syringe should always be made first. Instruments are, however, occasionally of great assistance, and sometimes absolutely necessary. To use them with safety the external auditory passage requires to be thoroughly illuminated; unless this can be affected, there is danger of producing more injury than might result from allowing the body to remain. A pair of rectangular forceps furnished with teeth will be found of great service for the removal of substances which admit of being grasped, as paper, cotton, etc. The principal risk in their use is the danger of pushing the body further into the canal. This can be avoided generally by fixing it with the pointed end of the curette, before grasping it with the forceps.

The curette and other instruments are sometimes used as levers, by making a fulcrum of the walls of the canal. This method of procedure should always be avoided. If the body is but a short distance in the meatus it can be removed more easily and with less risk than by this method. If the body is well advanced in the canal such a course can do no good, and may be of positive injury to the soft parts. Cases which seem to require the use of instruments in this manner, can be best treated by fixing the body with the curette, and then grasping it with the forceps as above described.

After the removal of foreign bodies there is generally considerable vascularity not only of the meatus, but of the membrana tympani. This is often due to the irritation produced by the foreign substances, but it is usually attributable to the efforts at removal. It is, however, of short duration, lasting frequently less than a day.

But little after-treatment will generally be required. In cases accompanied with considerable inflammation of the meatus, it may be necessary to use injections of tepid water. Should it show a tendency to become chronic in its character, the addition of a few gains of acetate of lead to the ounce of water will generally be found sufficient to arrest it.—*Boston Medical and Surgical Journal.*

